# Landscape determinants of human-elephant conflict in Assam, India: insights from two decades of spatial analysis

**DOI:** 10.7717/peerj.21082

**Published:** 2026-05-21

**Authors:** Athira N. G, Ramesh Kumar Pandey, Kalpana Roy, Ananya Dutta, Dheeraj Mittal, Parag Nigam, Anukul Nath, Bilal Habib

**Affiliations:** 1 Wildlife Institute of India, Dehradun, Uttarakhand, India; 2 Ministry of Envioronment Forest and Climate Change, Delhi, India; 3 Academy of Scientific and Innovative Reserach, Ghaziabad, Uttar Pradesh, India

**Keywords:** Asian elephant, Electrocution, Corridor, Fragmentation, Tea gardens, Village level prioritisation

## Abstract

Human-elephant conflict (HEC) presents an escalating conservation and socio-economic challenge across Assam, India, where a dense human population overlaps with critical elephant habitats. This study investigates human mortality patterns linked to HEC over 23 years (2000–2023), analysing 1,806 incidents, including 1,468 human fatalities and 337 human injuries. The data for this study were collected from 21 forest divisions of Assam and included detailed information (with gender specific details). Using spatial analyses, kernel density mapping, and generalised linear modelling (GLMs), this study identified key ecological and anthropogenic drivers of conflict. Conflict hotspots were predominantly located near fragmented forests, elephant reserves, and agricultural landscapes adjoining forests, with more incidents occurring during the monsoon and post-harvest seasons. Villages with limited or reduced forest connectivity and expanding built-up areas exhibited higher conflict intensity. Fragmentation metrics such as the Largest Patch Index and the Edge Density of the forest were significantly associated with the probability of HEC occurrence. Our findings highlight the importance of integrating ecological restoration, seasonal risk forecasting, and community-driven mitigation strategies such as buffer cropping and early warning systems. This study provides critical insights for prioritising high-risk areas and proposes practical interventions to promote coexistence between humans and elephants in a rapidly transforming landscape.

## Introduction

Human-elephant conflict (HEC) is a primary emerging concern in the field of conservation, characterised by direct and indirect associations between humans and elephants, which manifest in property damage, crop raiding, and human and elephant deaths (Sukumar, 2003). In India, HEC is an immense problem, with thousands of incidents causing enormous economic losses and deaths (Majumder, 2022). Mainly due to encroachment of forested habitat, expansion of agriculture, and fragmentation of migratory routes, which compel elephants into proximity to human habitations (Deb et al., 2022; Naha et al., 2019). Moreover, such encounters not only represent a threat to wildlife populations due to retaliatory killings but also place substantial socio-economic and psychological strains on local communities that depend on agricultural and natural resources for their sustenance (Köpke et al., 2024; Shaffer et al., 2019).

In India, elephants have always held an equal place with other animals in terms of cultural and religious value to the country, and they are revered for their representation of wisdom, strength, and good fortune (Krishna, 2010). Ancient texts and folklore reveal a deep respect for elephants, describing them as divine beings linked to Ganesha, the remover of obstacles and bringer of good fortune (Edgerton, 1931). Additionally, rural traditions have long regarded them as protectors of forests. Elephants in the rural areas were christened as protectors of the forests and formed an essential part of numerous rituals and functions (Fernando et al., 2008). This coexistence was made possible through contiguous and extensive forests that provided ample resources for elephants and minimised their need to enter human settlements (Sukumar, 2006). In the north-eastern part of India, Assam is well known for its rich biodiversity and extensive habitats for elephants. The rich variety of ecosystems in the State, ranging from the Brahmaputra Valley to grasslands and tropical forests, provides an ideal habitat for a substantial population of Asian elephants (*Elephas maximus*). With an estimated population of 5,828, the elephants of Assam represent more than a fair share of India’s elephant count (Piraisoodan, Arun Vignesh & Jayashree, 2024). The landscapes so offered have the required diverse ecological niches within which elephants could flourish, a fact that makes India host to more than 60% of the world population of Asian elephants (Baskaran et al., 2011).

The local communities in Assam have long experiences of living with elephants, a relationship best described as one of venerated fear (Gubbi, 2012). To vast populations of Assamese communities, the elephants stand for power, fortune, and the divine in the cultural and religious life. The population pressure, with the improved population of over 31 million in Assam (Government of Assam, 2011), surged; consequently, there were significant clearances of forests for urbanisation, infrastructure, and cultivation (Borah et al., 2005). Ever-increasing urbanisation and continuous habitat destruction due to deforestation and encroachment led to an increase in human-elephant interactions in Assam (Chartier, Zimmermann & Ladle, 2011; Kamdar et al., 2022). Due to habitat displacement, elephants are compelled to enter human-dominated areas in search of water and food, which frequently leads to fatalities. The expansion of monoculture plantations, particularly tea, which is one of the major crops in Assam, has also encroached into the habitats of elephants (Banerjee, Nayak & Sinha, 2024; Nad, Roy & Roy, 2022). Most often, these plantations have replaced biodiverse forests that provide a variety of food resources for elephants. Infrastructure development, including railways, roads, and dams, has also fragmented habitats by cutting elephant corridors and thereby isolating populations (Venkataraman, Tiwari & Ramkumar, 2017). These form barriers that exclude elephants from using their resources and concentrate them in areas of high human use, thus causing a competition for space (Venkataraman, Tiwari & Ramkumar, 2017). Expansion of human settlement has brought people and elephants into direct encounters, often forcing elephants to forage in the new settlements (Gubbi, 2012). Several studies across India have examined patterns of human–elephant conflict (HEC) using different approaches, including spatial hotspot mapping (Tripathy et al., 2021), spatio-temporal assessments in conflict-prone districts of Assam (Sangma et al., 2025), evaluations of mitigation techniques in West Bengal (Chakraborty & Paul, 2021), and community-based surveys around Kaziranga National Park (Pandey et al., 2024). While these studies provide valuable insights, most are restricted either to shorter time frames, localised areas, or specific thematic aspects such as mitigation or perceptions. In contrast, Assam, which harbours one of the largest elephant populations in India, still lacks a comprehensive long-term analysis of HEC that spans multiple forest divisions and integrates both ecological and demographic dimensions.

The current study aims to analyse the patterns of human mortality due to human-elephant conflict in Assam, identify influencing factors, and prioritise villages for targeted mitigation measures. The specific objectives are as follows: (a) understanding the patterns (both spatial and temporal) of human mortality due to HEC in Assam, (b) to examine the demographic patterns of human mortality resulting from human-elephant conflict, and (c) to prioritise villages with varying levels of conflict for implementing targeted mitigation measures. We hypothesise that human mortality due to human elephant conflict varies significantly across Assam, with higher mortality rates in areas closer to critical elephant habitats and corridors. We also expect that human mortality due to HEC has significant seasonal variation, with higher rates occurring during certain times of the year. Besides, specific demographic groups, such as age and gender, are expected to be disproportionately affected by HEC across different regions in Assam. We also expect that Human mortality rates increase in proximity to factors predicting human-elephant conflict, such as water sources, crop fields, and forest edges.

This study would enable the prioritisation of areas for targeted mitigation measures, contribute to a detailed understanding of HEC impacts, and support effective strategies to reduce human mortality and manage conflicts in Assam.

## Study area

**Figure 1:**
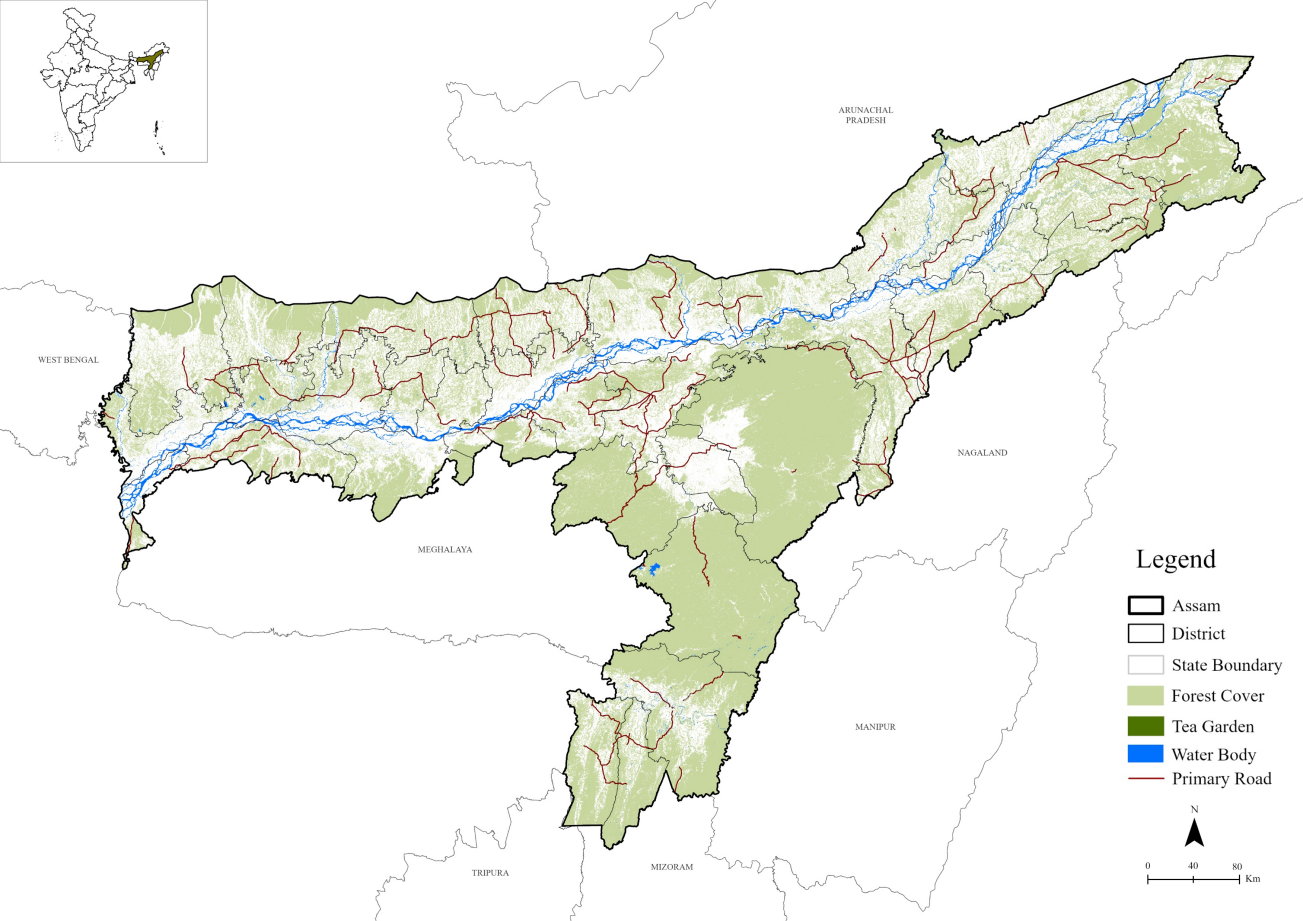
Map showing study area Assam, India. Map Source Credit: ArcGIS Living Atlas of the World (https://livingatlas.arcgis.com/en/home; ESRI 2023).

Assam lies in northeast India between latitudes 24° and 28°N and longitudes 89.5° and 96°E. Its northern neighbours are Bhutan and Arunachal Pradesh, while in the east lie Nagaland and Manipur. Meghalaya, Tripura, Mizoram, and Bangladesh surround it to the south, and West Bengal is its western neighbour. It covers an area of 78,438 km^2^, stretching from the Brahmaputra Valley in the north to the Barak Valley in the south, and includes the central districts around Karbi Anglong and Dima Hasao (Fig. 1). Elevations range from near sea level to hills rising to 1,965 m. The Brahmaputra River and its tributaries create a fertile plain ideal for agriculture. The annual average rainfall ranges between 2,000 and 3,000 mm annually, which falls during the rainy season in June–September.

A tropical monsoon climate is characterised by hot, humid summers and mild winters with heavy rainfall during the wet season. Assam is a State rich in biodiversity, with extensive nature reserves and national parks. Kaziranga, Manas, and Dibru-Saikhowa National Parks are home to endangered species such as the Indian rhinoceros, Bengal tiger, and Asian elephant. Concerning this, the previous review conducted shows that Assam’s forests contribute significantly to the State’s ecological landscape and biodiversity. They include the subtropical, semi-deciduous, and tropical rainforests, which cover a larger area of the State and provide habitat to various species (Easa, 2017). Assam’s forests are very valuable to its ecological landscape and biodiversity (Choudhury, 2004). These deciduous forests’ lower altitudes may host mixed flora and fauna, while the forested plains of the Brahmaputra valley are dynamic ecosystems with many herbivores and carnivores reported by different researchers. The Asian elephant needs to inhabit these kinds of forests because they afford adequate food and space (Sukumar, 2011). Elephants can maintain the health of forests through seed dispersal and, by creating flukes for the growth of new plants (Poulsen et al., 2021).

The ethnic mosaic of the State–Bodos, Karbi’s, Misings, and others contributes to the vibrant cultural tapestry through festivals such as Bihu and classical dance like Sattriya. Assamese language coexists with languages like Bodo and Karbi, presenting a linguistic diversity (Das, 2021). Traditional crafts like Assamese silk handloom weaving, bamboo & cane work have made significant contributions to the cultural identity and economy of the State. The most significant proportion of the population in Assam lives in rural areas and makes a living from agriculture. Indigenous communities like the Bodos and Karbis have traditionally drawn sustenance and parts of their culture from forests (Das, 2021). Loss of forest cover has entailed the displacement of these communities and reduced biodiversity, increasing human-wildlife conflicts, especially with the Asian elephant. This environmental degradation increased socio-economic challenges, making the continuity of traditional lifestyles associated with these communities challenging (De Silva et al., 2023).

Assam has been an agriculturally important producer of rice and tea due to the fertile plains of the Brahmaputra and Barak valleys, which are particularly suitable for cultivating these two crops. Tea gardens, more specifically from Jorhat and Dibrugarh, are immensely crucial for the State’s economy. Continuing deforestation has had adverse effects on agricultural productivity because of the depletion of soil fertility (Barooah, Phukan & Kalita, 2023). The literacy rate of Assam is now about 80.5% as of 2021, denoting that gaps still prevail between the urban and rural areas (Government of Assam, 2021).

## Methodology

### Collection of HEC occurrences

Information on HEC incidents was collected from 21 Divisional Forest Offices in Assam, spanning the years 2000 to 2023. The data encompassed details like the division name, village, incident dates, human fatalities, and injuries (with gender specifics). The forest divisions played a role in verifying the data, ensuring its accuracy. The verification process was carried out through visits to high-conflict villages that the Forest Department had consistently recorded in previous years. Since the frontline staff and guards working in these areas have first-hand knowledge of the frequency and intensity of conflict, their inputs were vital in confirming the accuracy and reliability of the data collected.

### Spatial distribution of HEC

The dataset included 1,806 instances of human fatalities and injuries caused by wild elephants over 23 years in Assam. It was segmented into 5-year intervals: 2000–2005, 2006–2010, 2011–2015, 2016–2020, and 2021–2023. Human fatalities and human injuries, gender, year, season, and division categorised the incidents. To illustrate spatial conflict hotspots, kernel density estimation maps were generated using ArcGIS with a 200-meter output cell size for precise geolocation. The severity of HEC was evaluated by examining the frequency of incidents across villages within the forest divisions. Villages were classified by conflict intensity: high (more than 20 incidents), medium (11–20), and low (1–10). The village boundaries were obtained from the ArcGIS Online, shapefile: Indian Administrative Layer 2024.

### Landscape fragmentation analysis

Land Use Land Cover (LULC) maps for Assam were developed using Landsat 5 TM and Landsat 8 OLI imagery (https://earthexplorer.usgs.gov/) for the years 2000, 2005, 2010, 2015, 2020, and 2024 , with a 30 m spatial resolution, and were used as the primary datasets. Preprocessing steps included atmospheric correction, cloud and shadow masking using the QA band, and delineation of the study area in Google Earth Engine (GEE). A supervised Random Forest (RF) classifier was applied to the multispectral imagery to categorise land cover into five classes: forest, water body, barren land, agriculture, and settlements. Ground truthing was performed using high-resolution Google Earth Pro imagery and visual interpretation of homogeneous areas, with a minimum of 250 samples per class. Seventy per cent of the samples were used for training and thirty per cent for validation. The RF-200 tree model produced classified maps for each year, which were then used to calculate land cover change between consecutive periods (Fig. S1).

Landscape fragmentation was analysed with FRAGSTATS (v4.2) to compute key landscape metrics. The input data from LULC maps were reclassified into forest and non-forest categories using the ArcGIS Spatial Analyst tool. Metrics like Patch Density (PD), Edge Density (ED), and Largest Patch Index (LPI) were calculated to capture landscape characteristics effectively. A 7 km moving window analysis, consistent with the average elephant movement patterns (Hassan et al., 2023), was used to produce continuous surfaces that represent both localised and broader spatial conflict patterns.

### Factors influencing human fatalities and injuries

To examine the factors influencing HEC incidents, we extracted variables such as distances to forests, croplands, urban areas, roads, waterways, railways, protected areas, and elephant reserves. Fragmentation metrics like Patch Density (PD), Largest Patch Index (LPI), and Edge Density (ED) were extracted to assess landscape structure and fragmentation in conflict zones. *A priori* hypothesis was made for all the variables (Table 1). These variables were derived from satellite imagery-based LULC layers created in the study, which were converted into vector formats. Conflict points were grouped into five intervals (2000–2005, 2006–2010, 2011–2015, 2016–2020, and 2021–2023) and overlaid on the corresponding LULC layers. For each interval, the LULC layer from the end year was used (*e.g.*, 2005 LULC layer for 2000–2005 conflicts). For conflicts within a specific year, the LULC layer from that closest year was used, allowing a systematic analysis of the relationship between conflict points and LULC changes over time. Using the “Generate Near Table” tool in GIS, the shortest distances between conflict points and these features were calculated. These distance values, along with fragmentation metrics, served as predictor variables, while human fatalities and injuries were the response variables. Generalised Linear Models (GLMs) were used for model selection with the “*MuMIn*” package in R (Version 1.3.1093). The models were constructed based on pre-set hypotheses, ensuring the inclusion of variables relevant to HEC (Table 1). Before building the models, all variables were z-transformed by calculating the mean and standard deviation of each column and then normalising the values. Pairwise correlations were visualised using a correlation matrix, and multicollinearity was assessed with the Variance Inflation Factor (VIF), excluding variables with VIF > 5. Models were ranked using the Akaike Information Criterion (AIC) (Burnham & Anderson, 2002). Model selection was conducted through univariate analyses assessing the significance of each predictor. We then developed multiple candidate models and evaluated them using Akaike Information Criterion (AIC), retaining models with ΔAIC ≤ 2 (Burnham & Anderson, 2002). The final model was selected based on the lowest AIC value and retained only significant predictors, ensuring an optimal balance between explanatory power and parsimony. This approach allowed us to identify key spatial and landscape determinants of HEC and provided critical insights for targeted mitigation strategies. Each HEC event involving human fatalities was coded as 1, while pseudo-points (areas without conflict) were randomly assigned a value of 0. Pseudo-points were generated near conflict zones within the study area, ensuring spatial separation while retaining contextual relevance. Specifically, the pseudo-points were created at least 1 km away from actual conflict points to maintain appropriate spatial separation, minimising spatial autocorrelation while ensuring representative sampling of the landscape. Earlier studies on HEC modelling have used similar distances, showing that a 1 km buffer maintains spatial independence without losing ecological relevance (Sharma et al., 2020).

**Table 1:** *A priori* hypotheses on environmental and anthropogenic factors influencing human mortalities and injuries in Assam.

**Feature**	**Variable**	**Description & Source**	**A-Priori Hypothesis**
**Landcover**	Distance from Built-up (db)	Extracted from classified landcover; distance calculated using Near Table tool (ArcPro 3.0.0).	Higher proximity may reduce HEC due to increased human activity and habitat loss.
	Distance from Croplands (dc)	Distance from cropland calculated using Near Table tool (ArcPro 3.0.0).	Closer proximity increases HEC due to crop damage.
	Distance from Forest (df)	Distance from forest calculated using Near Table tool (ArcPro 3.0.0).	Closer proximity may increase HEC as elephants move into human settlements.
	Distance from Waterbodies (dw)	Distance from waterbodies calculated using Near Table tool (ArcPro 3.0.0).	Closer proximity increases HEC, especially in dry seasons.
	Distance from Tea Gardens (dtea)	Extracted from Roy et al. (2016) LULC classification; distance calculated using Near Table tool (ArcPro 3.0.0).	Closer proximity may increase HEC due to habitat loss, pesticides, and human activity.
**Anthropogenic**	Distance from Roads (dr)	Distance calculated using Near Table tool (ArcPro 3.0.0).	Closer proximity increases HEC due to habitat fragmentation and human activity.
	Distance from Protected Areas (dpa)	Shapefiles from Elephant Cell, WII; distance calculated using Near Table tool (ArcPro 3.0.0).	Conflict may rise near protected area edges due to human presence.
	Distance from Elephant Reserve (der)	Distance calculated using Near Table tool (ArcPro 3.0.0).	Increased elephant movement near reserves may lead to higher HEC.
**Fragmentation metrices**	Edge Density (ed)	Total length of landcover edges per unit area.	Higher edge density increases deaths due to more fragmentation and encounters
	Patch Density (pd)	Number of habitat patches per unit area.	High patch density increases deaths due to fragmented and risky landscapes.
	Largest Patch Index (lpi)	Size of the largest continuous habitat patch in the landscape.	Proximity to large patches reduces deaths due to better resources and less movement.

## Results

### Temporal pattern and seasonality

**Figure 2:**
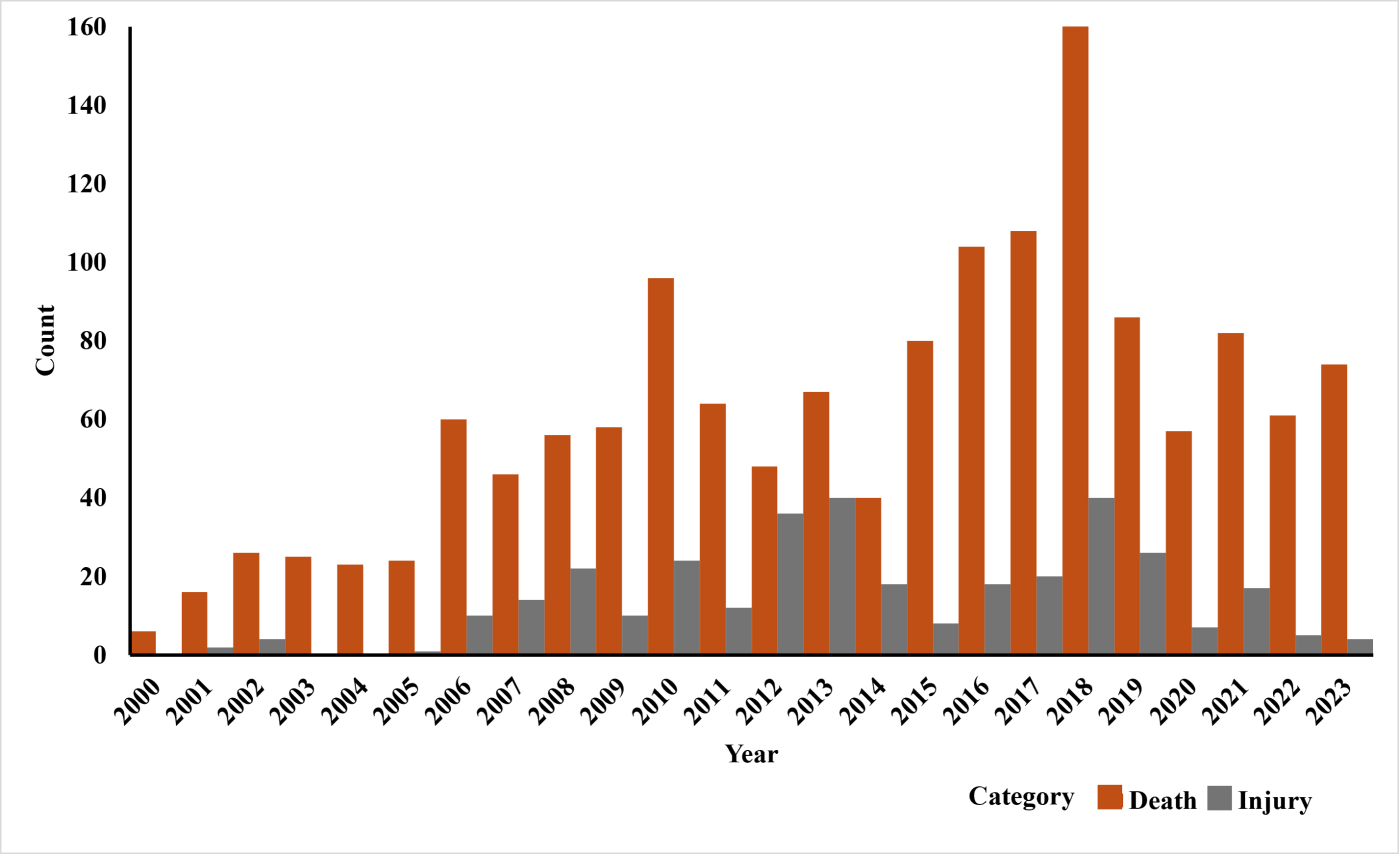
Trends in human fatalities and injuries due to human-elephant conflict in Assam (2000–2023).

**Figure 3:**
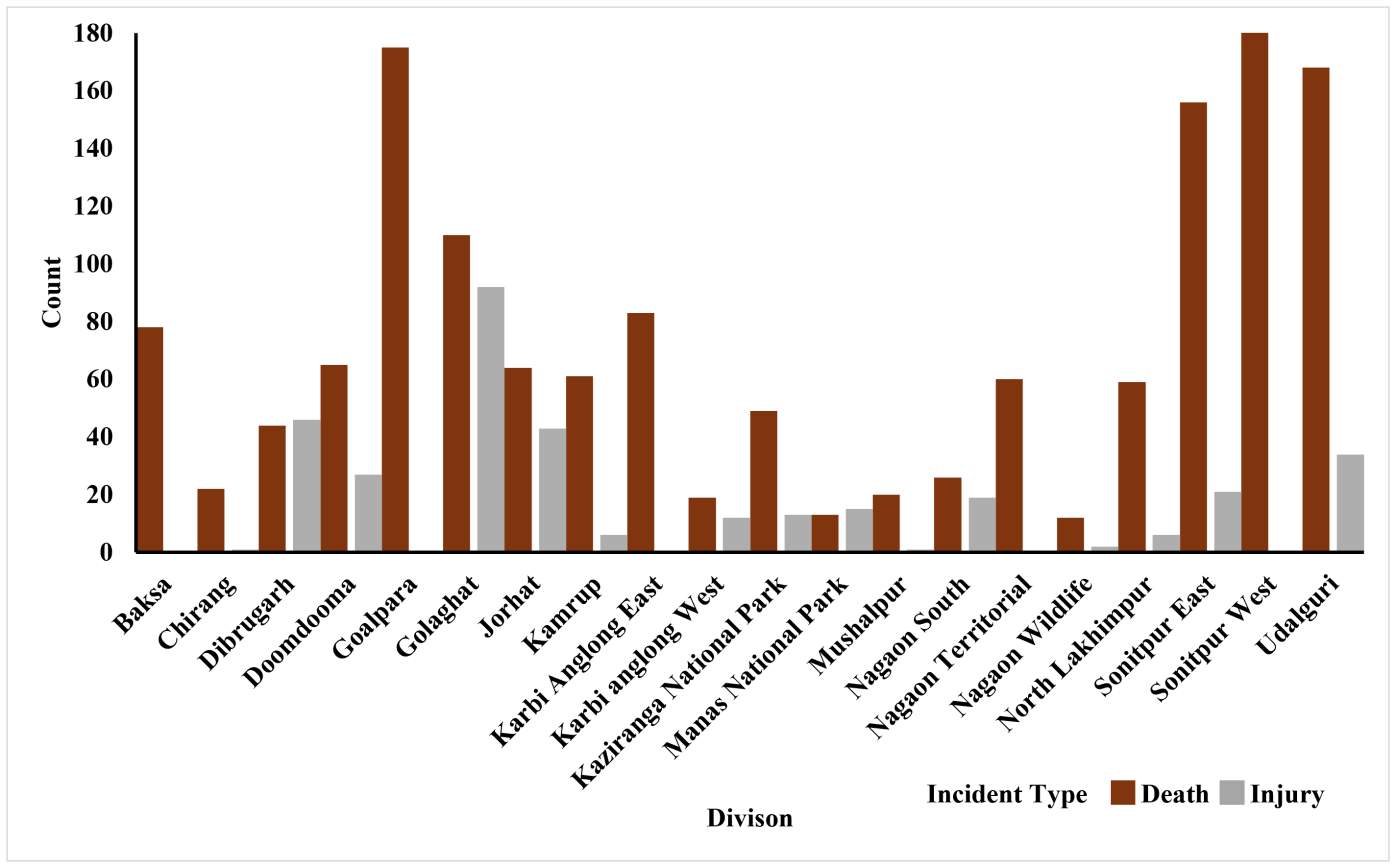
Division-wise distribution of human deaths and injuries resulting from human-elephant conflict in Assam (2000–2023).

**Figure 4:**
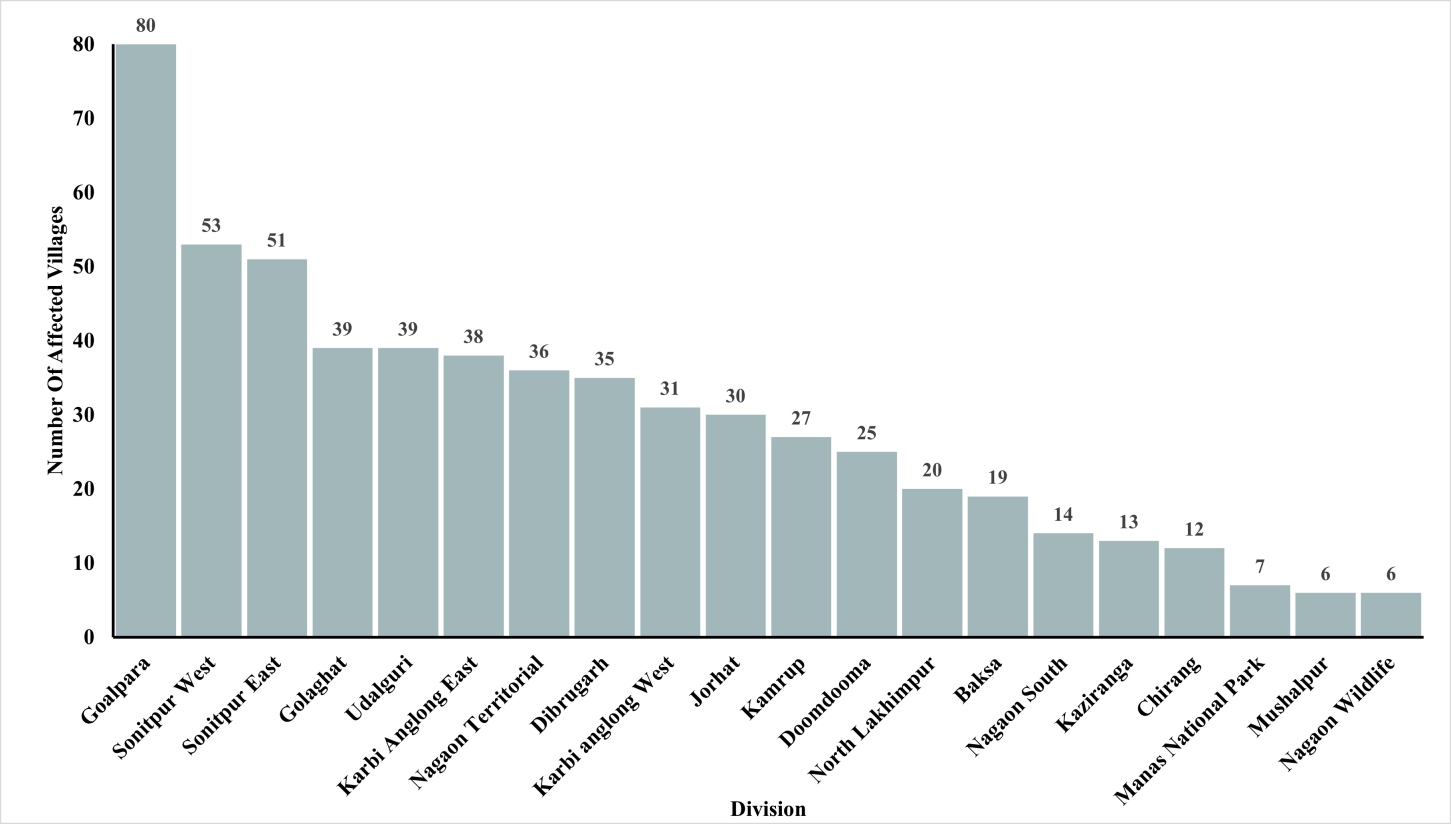
Division-wise distribution of villages affected by human-elephant conflict in Assam (2000–2023).

**Figure 5:**
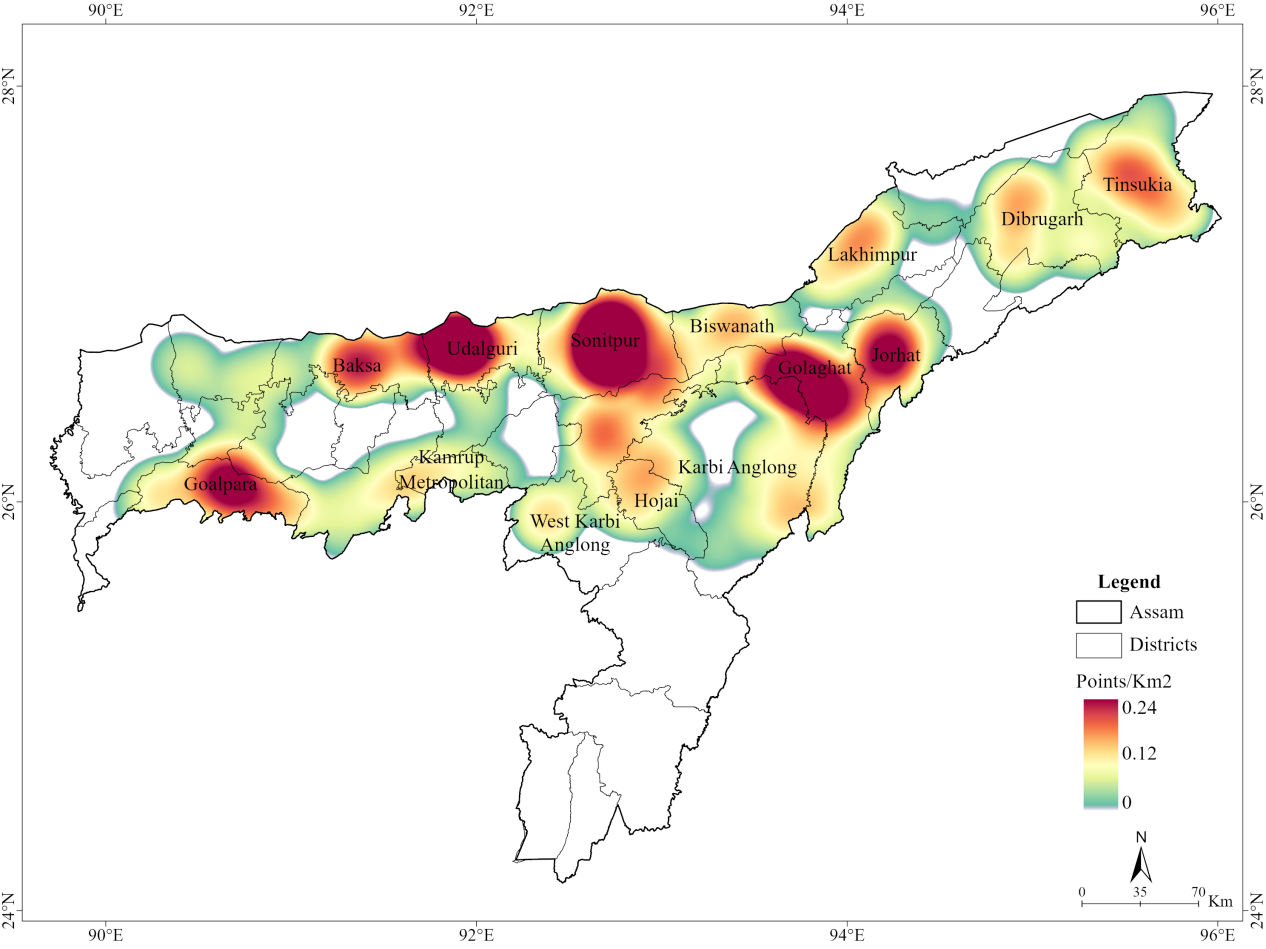
Hotspots of human deaths and injuries due to human-elephant conflict in Assam (2000-2023). Map Source Credit: ArcGIS Living Atlas of the World (https://livingatlas.arcgis.com/en/home; ESRI 2023).

**Figure 6:**
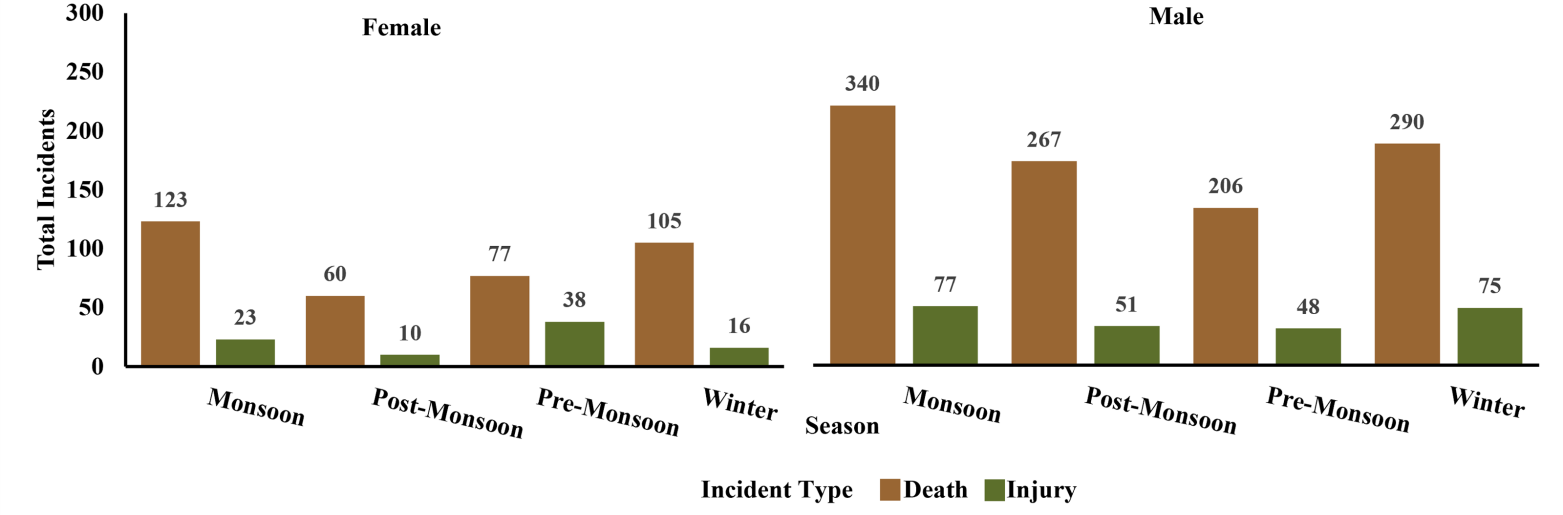
Seasonal variation in human deaths and injuries due to human-elephant conflict: gender-wise distribution across all seasons.

From 2000 to 2023, a total of 1,806 human-elephant conflict incidents were recorded in Assam, including 1,468 deaths and 337 injuries. The highest number of deaths was recorded in the year 2017 (Fig. 2). The seasonal distribution of incidents revealed the highest number of cases during the monsoon season. Incidents involving male victims were significantly higher across all seasons (*χ*^2^ = 17.751, *df* = 3, *p* = 0.00049) (Fig. 3). The division-wise analysis revealed that Sonitpur West (110 deaths, 92 injuries) recorded the highest number of incidents, followed by Goalpara (175 deaths), Udalguri (168 deaths, 34 injuries), Sonitpur East (156 deaths, 21 injuries), and Golaghat (110 deaths, 92 injuries) (Fig. 4). Over the years 527 villages were affected by human elephant conflict, Goalpara reported the maximum number of impacted villages (80), followed by Sonitpur West (53), Sonitpur East (51), and Udalguri (39) (Fig. 5). Kernel density estimation further highlights these divisions as hotspots for human-elephant conflict, with a high density of incidents concentrated in and around these areas (Fig. 6).

### Ecological and anthropogenic drivers of HEC

**Figure 7:**
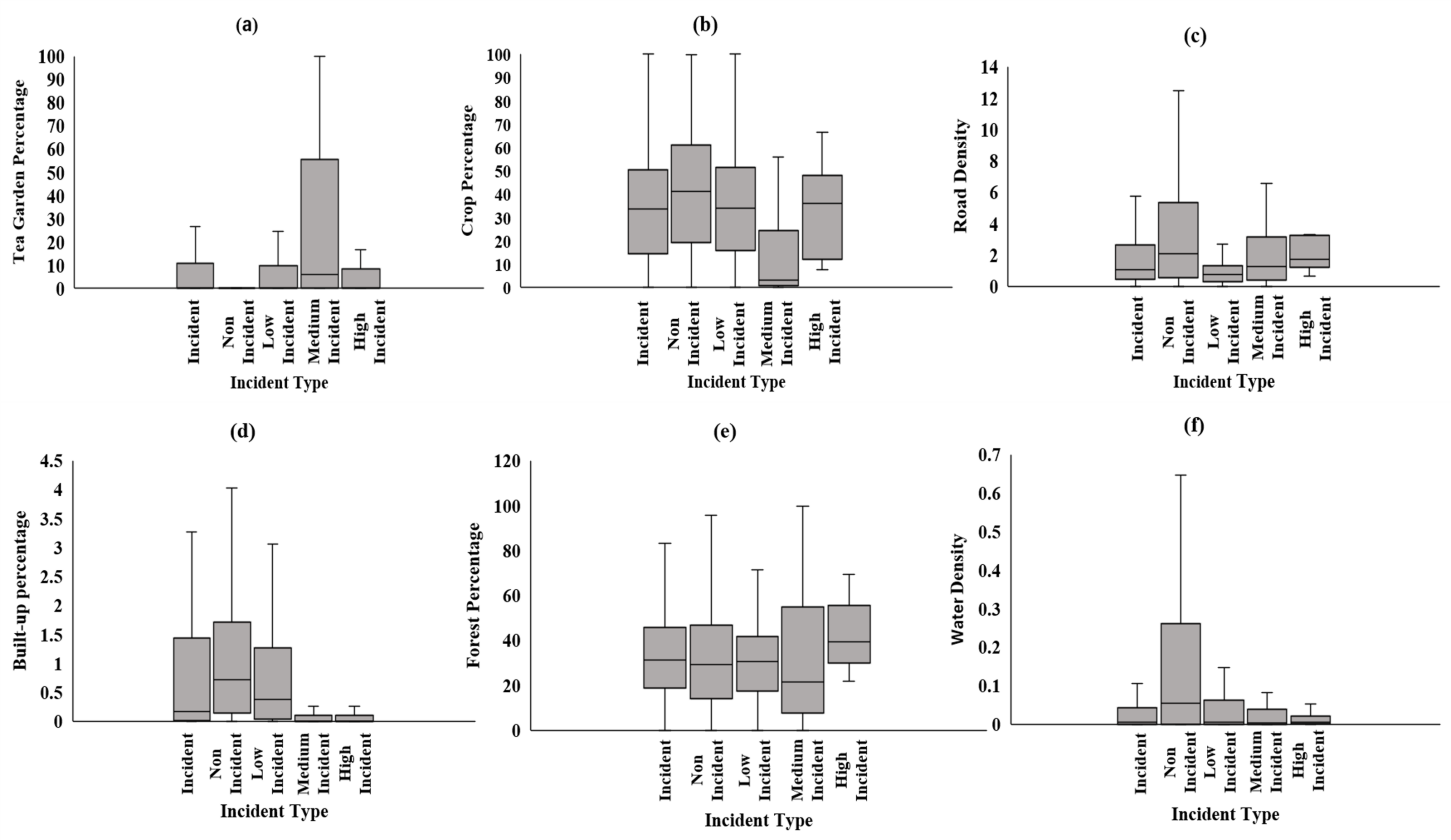
Spatial proximities of human-elephant conflict sites to key landscape features.

Proximity to natural and human-modified landscapes significantly influences human-elephant conflict probability. Conflict likelihood increased with proximity to water bodies (*β* = 0.37, *p* < 0.001), forests (*β* = −0.189, *p* = 0.05), croplands (*β* = −0.06, *p* = 0.33), and built-up areas (*β* = −0.43, *p* < 0.001). Incidents were more frequent near forests (*β* = -13.40, *p* < 0.001) and elephant reserves (*β* = −0.24, *p* < 0.001), and tea gardens (*β* =−0.08, *p* = 0.04), indicating substantial spatial overlap between elephants and humans in these areas. Conversely, conflict probability decreased with increasing edge density (*β* = 0.17, *p* < 0.001), patch density (*β* = −0.385, *p* < 0.001), indicating a negative association and the largest patch index (*β* = −0.531, *p* < 0.001) (Tables 2 and 3; Fig. 7).

**Table 2:** Summary statistics loglikelihood (LogL), degrees of freedom (df), Akaike Information Criteria (AICc), relative support for hypothesis (Δ AICc), Akaike weights (Wi) of candidate regression model explaining HEC in Assam.

Model	LogL	df	AICc	ΔAICc	Wi
dw + dr + dc + der + dpa + dta + lpi	−2169.67	8	4,355.39	0.00	0.50
global	−2169.31	9	4,356.68	1.29	0.26
dw + dr + der + dpa + dta + lpi + df	−2171.42	8	4,358.88	3.50	0.09
lpi + dw + dr + df + dc + der + dpa	−2171.70	8	4,359.45	4.06	0.07
der + dpa + dta + lpi + dw + dr	−2172.76	7	4,359.56	4.17	0.06
dw + dr + der + dta + lpi	−2175.20	6	4,362.43	7.05	0.01
dpa + dta + lpi + dw + dr	−2175.48	6	4,362.99	7.61	0.01
dw + dr + der + lpi	−2177.69	5	4,365.40	10.01	0.00
dta + lpi + dw + dr + df + dc	−2180.63	7	4,375.28	19.90	0.00
dw + dr + df + dc + der + dpa + dta	−2187.65	8	4,391.35	35.96	0.00
dw + dr + df + dc + der + dpa	−2194.53	7	4,403.09	47.70	0.00
dw + dr + df + dc + der	−2198.04	6	4,408.11	52.73	0.00
dr + der + lpi	−2206.11	4	4,420.23	64.84	0.00
Null	−2397.90	1	4,797.80	442.42	0.00

**Table 3:** Parameter estimates effect (*β*), standard errors (S.E), and probabilities of ecological and anthropogenic variables in determining the human elephant conflict in Assam.

Estimate	Beta coefficient (*β*)	Z value	*P* value	Significance
Intercept	0.009	0.23	0.82	
Distance to waterbodies (dw)	0.344	7.65	0.00	^***^
Distance to roads (dr)	−0.743	−9.71	<2e−16	^***^
Distance to crops (dc)	−0.150	−2.45	0.01	^*^
Distance to elephant reserves (der)	−0.106	−2.36	0.02	^*^
Distance to protected areas (dpa)	−0.102	−2.33	0.02	^*^
Distance to tea gardens (dtea)	−0.090	−2.18	0.03	^*^
Largest patch index (LPI)	−0.279	−5.97	0.00	^***^

**Notes.**

* (Significant) 0.01 < *p* < 0.05.

*** (Very Highly Significant) *p* < 0.001.

### HEC village level

**Figure 8:**
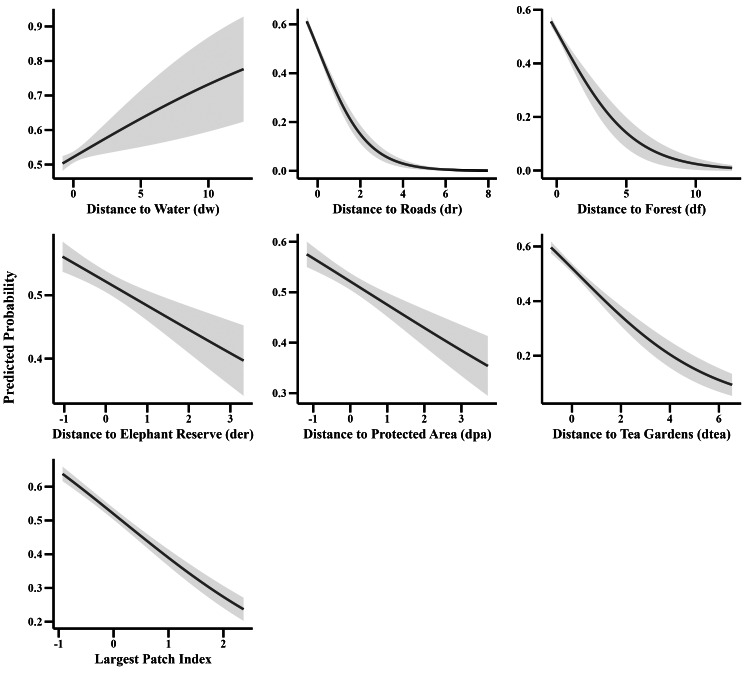
Predicted probability of human mortality due to human-elephant conflict in Assam based on environmental and landscape variables.

Our village-level analysis reveals distinct patterns across various factors associated with HEC in Assam. Forest percentage is relatively consistent across all categories; high-conflict villages tend to have smaller and more fragmented patches. Built-up areas are almost negligible in high-conflict villages, whereas medium-conflict villages show the highest built-up percentages, followed by low-conflict and non-conflict areas. Road density is lowest in high-conflict villages, with non-conflict areas having the highest density, indicating better infrastructure in regions with fewer conflicts. Tea garden percentage in medium-conflict villages having the highest concentration, while high-conflict villages have relatively lower tea garden percentages. Crop percentage is stable across categories, though medium- and high-conflict villages exhibit slightly lower percentages. Water density is highest in non-conflict villages, while high-conflict villages show the lowest density (Figs. 8A–8F). A *post hoc* test was conducted for each variable, with water, tea gardens, road, and built-up identified as significant variables (Table 4).

## Discussion

This study provides an ecological perspective on the drivers of HEC in Assam, highlighting how the deterioration of natural systems and species responses to anthropogenic pressures contribute to conflict. Seasonal patterns emerge as a significant driver of conflict, with the monsoon season witnessing heightened incidents of human fatalities and injuries. During this period, increased agricultural activities coincide with the seasonal movements of elephants. This convergence during the monsoon creates ecological overlap zones, which increase the probability of human-elephant interaction and lead to escalated conflict. This seasonal trend aligns with Africa, where elephants are similarly attracted to agricultural land during the rainy season, leading to escalated conflict with local communities (Blake et al., 2008). The seasonal convergence of elephants into human-dominated landscapes during the monsoon season in Assam reflects a broader pattern observed across India. In Tamil Nadu, HEC incidents peaked during the southwest monsoon, particularly in agricultural landscapes such as Coimbatore and the Nilgiris, where crop availability and reduced natural forage during the rainy season compelled elephants to enter human settlements (Shameer et al., 2024). Comparable trends were observed in Karnataka, where the highest levels of HEC occurred during the monsoon months due to the seasonal availability of crops and limited wild forage (Gubbi, 2012). Similarly, in Odisha, crop-raiding incidents peaked during the monsoon and winter seasons, with male elephants predominantly responsible (Naha et al., 2019). However, unlike Tamil Nadu, where open and accessible agricultural landscapes intensify conflict, Assam’s dense forest cover and challenging terrain may restrict elephant movement to some extent, thereby moderating—but not eliminating—the seasonal surge in human–elephant encounters.

**Table 4:** Kruskal–Wallis’s test results for different land-use variables in relation to incident type. Dunn’s *post-hoc* test: performed for significant results, and adjusted *p*-values.

**Variable**	**Kruskal–Wallis (*χ*^2^)**	**df**	***p*-value**	**Significance**	**Significant *Post-hoc* comparisons**	**p-adj**	**p-adj. significance**
Forest	4.8554	3	0.18	Not significant	–	–	–
Water	64.801	3	0.00	^***^	Incident *vs* Low Incident (*p* = 2.60e−14)	0.00	^****^
Road	17.89	3	0.00	^***^	Incident *vs* Low Incident (*p* = 0.000332)	0.00	^***^
Builtup	6.7863	3	0.08	Not significant	–	–	–
Crop	11.21	3	0.01	^*^	Incident *vs* Medium Incident (*p* = 0.00531)	0.03	^*^
TeaGarden	18.674	3	0.00	^***^	Incident *vs* Low Incident (*p* = 0.0277)	0.03	^*^

**Notes.**

* (Significant) 0.01 < *p* < 0.05.

** (Highly Significant) 0.001 < *p* < 0.01.

*** (Very Highly Significant) *p* < 0.001.

**** (Extremely Significant) *p* < 0.0001.

Landscape fragmentation is identified as a critical factor influencing the frequency and intensity of conflict in Assam. Proximity to fragmented forest patches, characterised by high edge density and patch density, contributes to the creation of conflict hotspots. Fragmented habitats often disrupt elephant movement and force elephants to venture into agricultural and settlement areas in search of food and water. The loss of functional elephant corridors, which are critical for maintaining habitat connectivity, exacerbates this issue. Assam has 12 identified elephant corridors, but rapid land-use changes and infrastructure development threaten their effectiveness, forcing elephants into human-dominated areas and increasing human mortality rates (Pandey et al., 2024). These corridors, if protected, could reduce conflict by allowing safe movement between habitats. Districts such as Sonitpur East and Goalpara, exhibiting protected areas, agricultural fields, and fragmented forests, were identified as major conflict zones. These results align with previous studies that have emphasised the role of habitat fragmentation in exacerbating HEC (Sukumar, 2006; Talukdar, Choudhury & Ahmad, 2024).

At the village level, the analysis reveals that high-incident villages exhibit narrower forest cover ranges, suggesting that habitat degradation and reduced forest connectivity are key contributors to conflict. Conversely, non-incident villages tend to have more contiguous forest cover, providing refuge for elephants and reducing the likelihood of encounters with humans. Built-up areas emerge as significant anthropogenic drivers of conflict. Villages with higher percentages of built-up areas are associated with medium- and high-conflict levels, whereas non-incident villages, characterised by minimal urbanisation, benefit from reduced human-elephant interactions. The expansion of built-up areas near elephant habitats often overlaps with traditional elephant corridors, intensifying conflict potential.

Additionally, railway and road infrastructure further influence conflict dynamics, as transport routes bisect key elephant habitats, leading to frequent accidents. Assam and West Bengal, in particular, report some of the highest railway-related elephant deaths, making it imperative to implement mitigation measures such as overpasses, underpasses, and speed regulation zones (Pandey et al., 2024). Transport infrastructure, such as roads, further influences conflict dynamics. While roads in non-incident areas act as barriers to elephant movement, their lower densities in high-conflict zones reflect less developed, remote areas where elephants have greater access to their former habitats. The role of tea gardens, a dominant land-use type in Assam, acts as a transitional zone between forests and human settlements. Tea gardens with small vegetation patches provide forage and cover for elephants, but simultaneously increase the likelihood of human-elephant encounters. Villages with significant tea garden coverage, particularly medium-incident villages, exhibit heightened conflict levels. Tea gardens often serve as buffer zones, yet their proximity to built-up areas and reduced forest connectivity complicates the coexistence of humans and elephants. These findings align with a study that highlighted the impact of monoculture plantations on elephant movement patterns and conflict escalation. Tea gardens, while beneficial for local economies, pose unique challenges in mitigating HEC (Madhusudan et al., 2015), especially in areas like Golaghat and Chirang, where elephant corridors overlap with human settlements. Water availability also plays a critical role in shaping conflict intensity. Villages with higher water densities tend to experience fewer incidents of HEC, as access to sufficient water sources reduces interaction with humans.

In contrast, high-conflict villages with limited water availability are more prone to encounters, as elephants are driven into human settlements in search of water. This pattern supports findings from other studies that emphasise the importance of water resources in mitigating human-wildlife conflicts (Gubbi et al., 2014). Between 2009 and 2020, human-elephant conflicts led to an average of 450 human deaths annually across India, with the northeast region being one of the most affected. The Indian government has recognised this issue, allocating an annual average of USD 4.79 million as compensation for property losses (Pandey et al., 2024).

These findings emphasise the urgent need for improved corridor protection, regulated infrastructure development, and community-based mitigation efforts to reduce human mortality. High-conflict villages have a low percentage of built-up areas, whereas non-incident areas tend to exhibit higher built-up areas, contrasting with the study in Keonjhar Forest Division, which showed that areas with greater human settlement near elephant habitats experienced more frequent conflicts (Tripathy et al., 2021). According to our study, these villages fall near the elephant corridors, as listed in Table 5. The factors influencing HEC further corroborate the influence of ecological and anthropogenic factors on HEC. Areas farther from water sources are more susceptible to fatal encounters, consistent with a study indicating that water scarcity drives elephants to travel long distances, increasing their interaction with human settlements (Leimgruber et al., 2003). This pattern reflects the broader ecological tendencies of elephants, which exhibit a strong dependency on water sources, particularly during the dry season when water availability becomes a critical determinant of their movement and habitat use. In contrast, the negative associations with roads, crops, elephant reserves, protected areas, and tea gardens suggest that human fatalities are more likely in areas with higher elephant activity or movement. The reduced risk of conflict near roads may be attributed to greater human presence and surveillance, aligning with findings from earlier studies (Sukumar, 2006).

**Table 5:** The following key elephant corridors, as identified in the “Project Elephant, Ministry of Environment, Forest and Climate Change, Government of India (2023)” report are critical for mitigationg human-elephant conflict.

Connectivity	Corridor name	Adjacent villages
Kaziranga–Karbi Anglong	Panbari Corridor	Nambar Lang Protham F V, Sam Singnar
Kaziranga–Hojai	Haldhibari Corridor	Hojai, Nagaon, Natum Salona Tea Estatee
Kaziranga–Nagaon	Hatinadi Corridor	Nagaon, Athubhanga, Nambar Lalung Gaon
Kaziranga–Golaghat	Kalapahar–Doigrung Corridor	Golampatty, Duborani, Grant
Kaziranga–Tezpur	Charduar–Singri Hill Corridor	Tezpur, Likhak Gaon, Gor Mara Gaon
Kaziranga–Doom Dooma	Kotha Buridehing Corridor	Doom Dooma, Digboi, Kherbari
Kaziranga–Jorhat	Kanchanjuri Corridor	Jorhat, Kathalguri Teas Etate, Holongpar
Kaziranga–North Lakhimpur	Kukurakata–Bagser at Amguri Corridor	North Lakhimpur, Fenkhati Gaoni, Paikan Pt II
Kaziranga–Sonitpur	Charduar–Singri Hill Corridor	Amaribari T E, Ambari, Phumen Ingti

Meanwhile, proximity to crops, protected areas, and tea gardens tends to increase conflict risk, likely because elephants are drawn to food resources and expand their natural range (Hoare, 1999). The relevance of the Largest Patch Index (LPI) highlights the role of landscape fragmentation in intensifying human-elephant conflict. Lower LPI values, which signify more fragmented habitats, are linked to increased conflict—supporting earlier research that shows how fragmented landscapes disrupt traditional elephant corridors and elevate the chances of human-elephant encounters (Goswami, Vasudev & Oli, 2014).

Landscape metrics such as edge density, largest patch index, and patch density were identified as significant predictors of conflict. Fragmented forest patches with high patch density are strongly correlated with increased conflict frequency, underscoring the critical role of habitat fragmentation. Similarly, built-up area coverage is positively associated with conflict, particularly in medium-conflict villages. These results are consistent with the findings of the importance of maintaining contiguous forest patches to reduce the risk of HEC (Leimgruber et al., 2003).

## Conclusion

**Figure 9:**
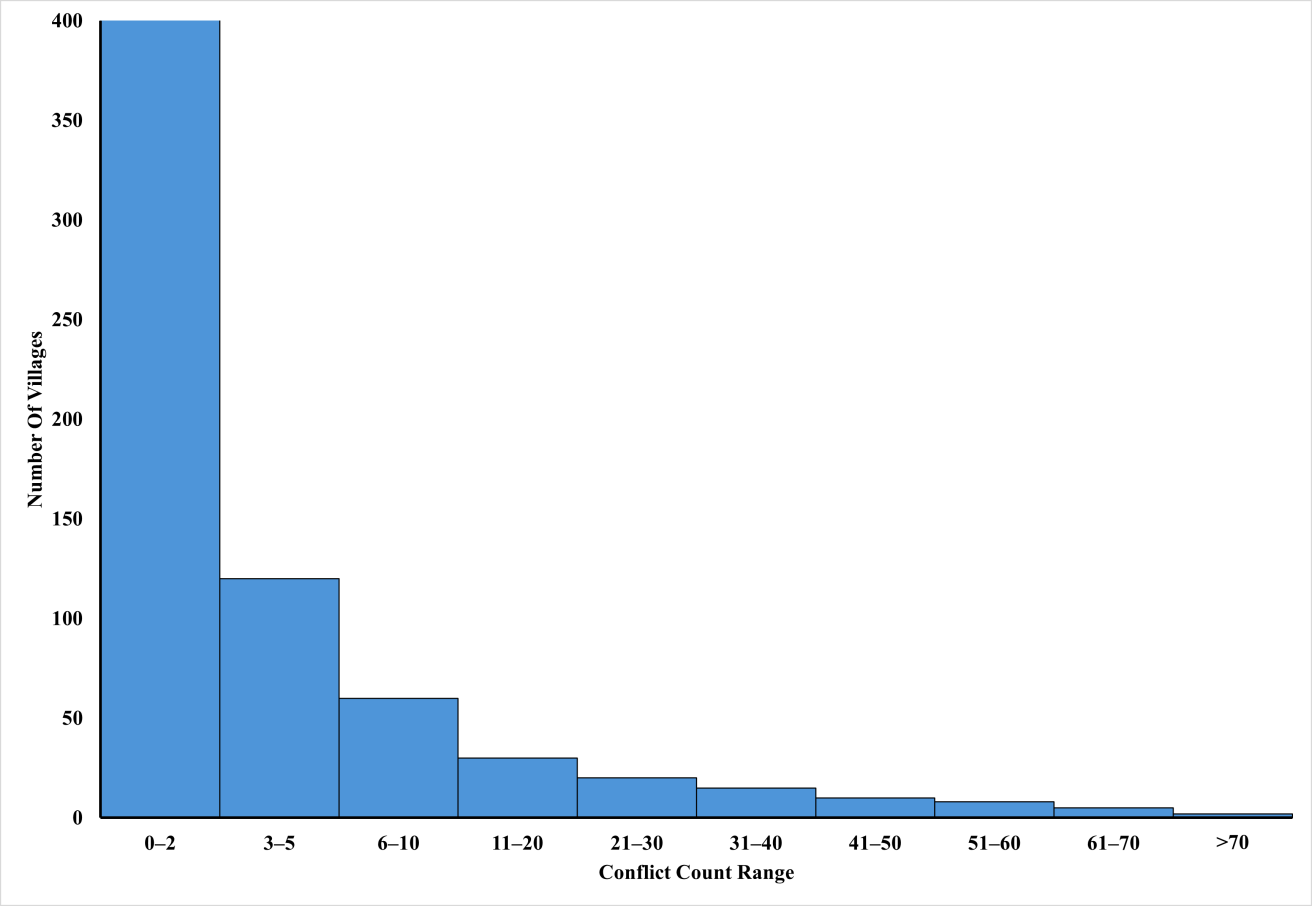
Distribution of human-elephant conflict incidents across villages based on conflict intensity in Assam.

This study provides a comprehensive analysis of HEC in Assam, revealing its complex and multifaceted nature, driven by the interplay of seasonal variations, ecological changes, and anthropogenic pressures. Conflict incidents were concentrated in a few villages, while most others experienced only a limited number of events over the past two decades (Fig. 9) High-conflict villages like Likhak Gaon (73 incidents), Jorhat (41 incidents), Ambari (40 incidents), Uttar Dimakuchi & Jogigaon (30 incidents), Gor Mara Gaon (28 incidents), Golampatty & Nagaon (26 incidents) and Kathalguri (24 incidents) in Assam are marked by habitat fragmentation, increasing urbanisation, and limited water resources, which exacerbate conflicts between humans and elephants. The findings highlight the urgent need for integrated and context-specific mitigation strategies that address the root causes of these conflicts. Effective mitigation measures in Assam have focused on restoring forest connectivity, regulating urban expansion, and improving water accessibility in conflict-prone regions. Installing low-voltage, elephant-friendly hanging electric fencing (5–6 KV) along village peripheries can deter elephants without causing harm. For long-term effectiveness, proper maintenance and community engagement in monitoring these fences are essential. Additionally, community-based initiatives, such as awareness campaigns, volunteer programs, and training local communities on coexistence strategies, have shown promising results in minimising human-elephant encounters. In contrast, less effective measures often include insufficient compensation schemes, which are unable to address the underlying socio-economic drivers of conflict, and top- down policies that lack community involvement in plan implementation. Advanced warning systems, such as infrared trip wire alarms, can detect elephant presence and alert communities in real time. Community-based elephant alert networks using mobile apps (*e.g.*, Gaj Yatra, Hathi Mirta, Hathi Alert) and radio messaging systems can help disseminate real-time warnings, reducing the risk of direct encounters. Cultivation of species like chilli (*Capsicum spp*.), ginger (*Zingiber officinale*), garlic (*Allium sativum*) and citrus fruits are unpalatable to elephants and can serve as buffer crops around high-risk farmlands.

 Given the varied success of different strategies across Assam’s diverse regions, it is crucial to adopt a tailored approach for high-conflict divisions. Strategies that incorporate both ecological restoration and community engagement, along with targeted land-use planning and seasonal monitoring, can significantly mitigate HEC. Creation of rapid response team and speedy compensation and ex-gratia are also play an important role in reducing hospitality and gaining community cooperation in meeting these challenges. Collaboration between local communities, wildlife authorities, and environmental organisations is essential to implement these measures effectively. Combining ecological restoration, community engagement, and policy interventions, must be employed to address the challenges of human-elephant conflict in Assam. By learning from existing research and adopting proven mitigation practices, we can foster a sustainable and peaceful coexistence between humans and elephants in high-conflict areas.

##  Supplemental Information

10.7717/peerj.21082/supp-1 Supplemental Information 1 Raw Data

10.7717/peerj.21082/supp-2 Supplemental Information 2 Supplementary Information

10.7717/peerj.21082/supp-3 Supplemental Information 3 Code

## Additional Information and Declarations

### Data Availability

The following information was supplied regarding data availability: The raw data and code are available in the Supplemental Files.
