# Supplementary material for: Landscape determinants of human-elephant conflict in Assam, India: insights from two decades of spatial analysis
Source: PeerJ. 2026 May 21;14:e21082. doi: 10.7717/peerj.21082 (PMC13198851; doi:10.7717/peerj.21082)
Supplement: Supplemental Information 2 [file peerj-14-21082-s002.docx]

**Supplementary**


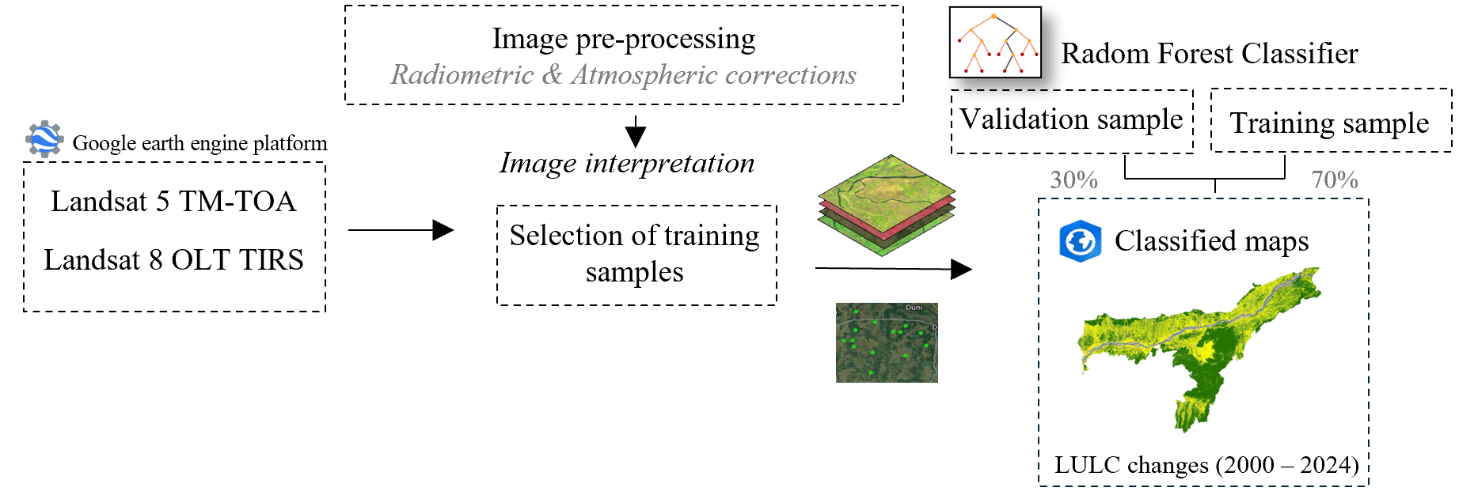


Supplementary Figure 1 Flowchart of methodology followed for LULC classification for Assam, India.


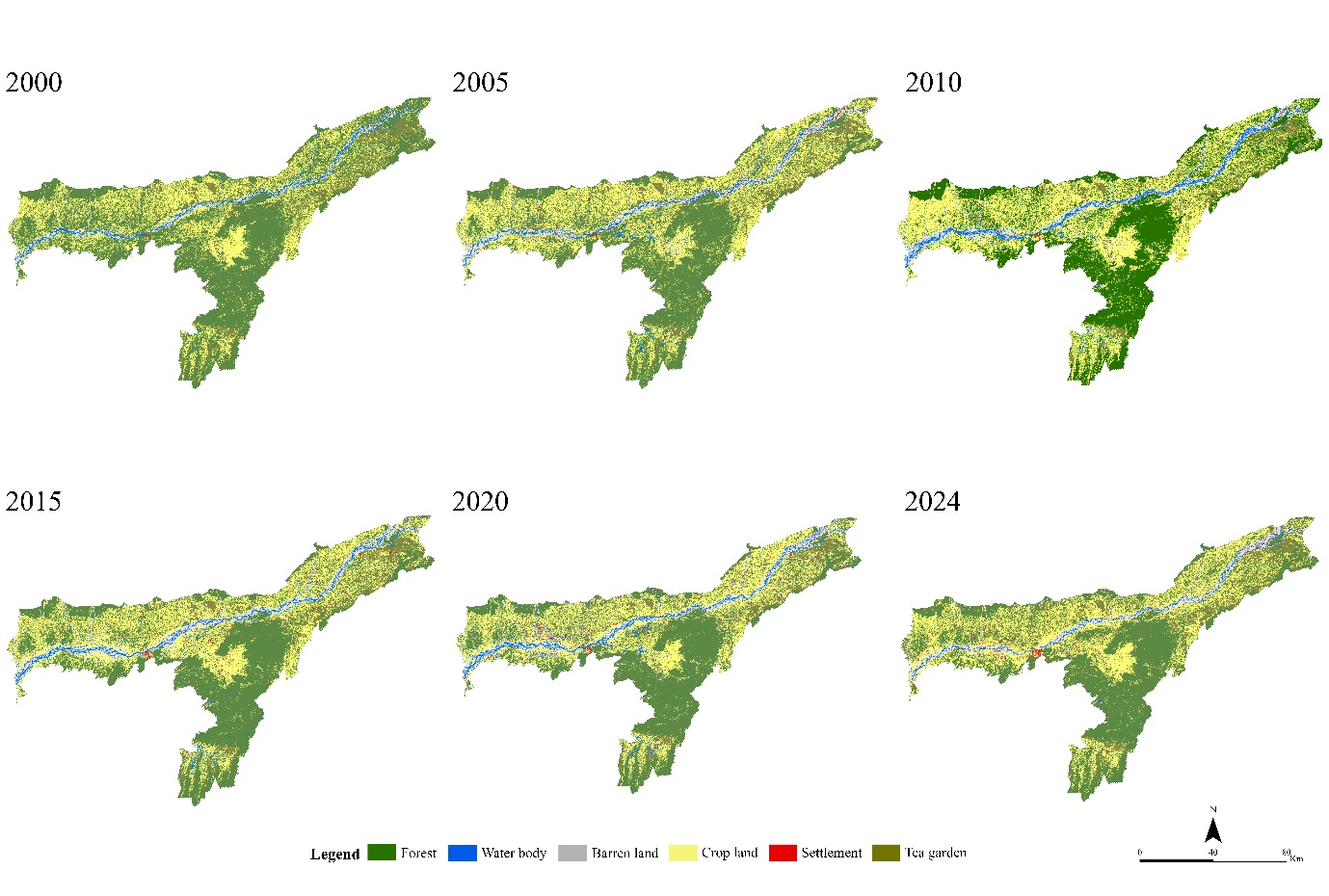


Supplementary Figure 2 Land use Land cover maps (2000-2024) of Assam, India.


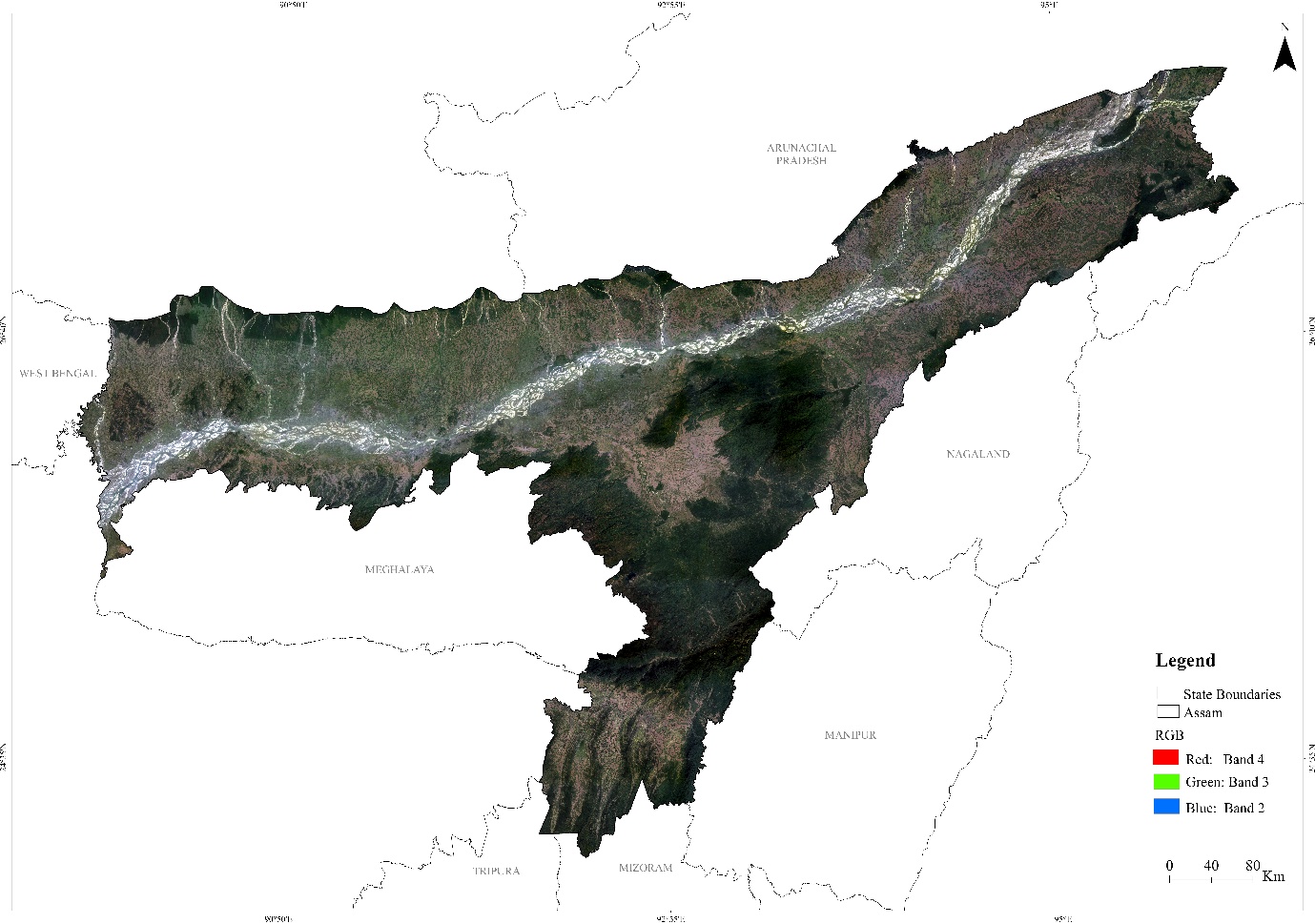


Supplementary Figure 3 Landsat 8 Satellite image in True colour combination of Assam , India.
